# A Comprehensive Analysis of Conventional Acupuncture and Pharmacological Approaches for Cardiac Arrhythmias: An Umbrella Review

**DOI:** 10.19102/icrm.2024.15055

**Published:** 2024-05-15

**Authors:** Tamam Mohamad, Mahima Khatri, Satesh Kumar, Maneesh Kumar, Aakash Kumar, Giustino Varrassi, Poonam Bai, Arjan Dass, Fnu Sapna, Alina Sami Khan, Abdul Ahad Syed, Areeba Maryam, Abdul Rehman Shah Syed

**Affiliations:** 1 Department of Cardiology, Wayne State University/Detroit Medical Center, Detroit, MI, USA; 2 Department of Medicine, Dow University of Health Sciences, Karachi, Pakistan; 3 Department of Medicine, Shaheed Mohtarma Benazir Bhutto Medical College, Lyari, Karachi, Pakistan; 4 Department of Medicine, Ghulam Muhammad Mahar Medical College, Sukkur, Pakistan; 5 Department of Anesthesiology, Paolo Procacci Foundation, Rome, Italy; 6 Department of Medicine, Penn State Health Milton S. Hershey Medical Center, Hershey, PA, USA; 7 Department of Medicine, Willis-Knighton Health System, Shreveport, LA, USA; 8 Department of Medicine, Liaquat National Hospital and Medical College, Karachi, Pakistan; 9 Department of Medicine, Rawalpindi Medical University, Rawalpindi, Pakistan

**Keywords:** Acupuncture, anti-arrhythmic drugs, cardiac arrhythmias, comparative effectiveness, prevention

## Abstract

With a global incidence of approximately 3.4% and an annual mortality rate of 3.7 million, cardiac arrhythmias (CAs) are a pressing global health issue. Their increasing prevalence, especially among older people, is intensifying the challenge for health care systems worldwide. This study aims to compare the safety and effectiveness of acupuncture and pharmacological treatments for CAs, addressing critical gaps in understanding optimal therapeutic approaches. A search of PubMed, EMBASE, and the Cochrane database of systematic reviews was performed to identify data compiled through September 2023 for this umbrella review. Randomized controlled trials (RCTs) as the foundation for meta-analyses and peer-reviewed systematic reviews were the primary focus of the literature search. The Grading of Recommendations Assessment, Development, and Evaluation method was used to assess the overall certainty of the evidence, whereas AMSTAR 2 and the Cochrane Collaboration tool were used to evaluate the quality of the included reviews. Following a comprehensive review, three systematic analyses of 27 RCTs were integrated. Acupuncture led to a slightly greater reduction in the recurrence rate of paroxysmal supraventricular tachycardia (SVT) compared to standard pharmaceutical therapy (risk ratio [RR], 1.06; 95% confidence interval [CI], 0.88–1.27; I^2^ = 56%; *P* = .55), although the difference was not statistically significant. In contrast, acupuncture significantly outperformed pharmacological treatment in the context of ventricular premature beats (VPBs) (RR, 1.16; 95 CI, 1.08–1.25; I^2^ = 0%; *P* < .0001). The reduction in paroxysmal atrial fibrillation (AF)/atrial flutter was increased with acupuncture, albeit without statistical significance (RR, 1.12; 95% CI, 0.88–1.42; I^2^ = 0%; *P* = .36). Acupuncture also led to a greater reduction in heart rate (HR) compared to pharmaceutical treatment despite notable heterogeneity and a lack of statistical significance (mean difference, −1.55; 95% CI, −41.37 to 38.28; I^2^ = 99%; *P* = .94). Adverse events were effectively managed, affirming the favorable safety profile of acupuncture. Our study suggests that acupuncture leads to a greater reduction in the recurrence rates of VPBs, AF, and atrial flutter but not significantly so in paroxysmal SVT or post-treatment HR. While promising for specific arrhythmias, the varying effectiveness of acupuncture underscores the need for further research and clinical assessment to determine its precise role and suitability in managing particular cardiac conditions.

## Introduction

Cardiac arrhythmias (CAs) refer to cardiac disorders marked by abnormal ventricular contractions.^1^ With a global incidence of approximately 3.4%, CAs contribute to an annual mortality rate of about 3.7 million.^2^ The increasing CA prevalence, especially among older people, poses a significant health care challenge. Effectively managing CAs is crucial due to their substantial impact on morbidity and mortality.^3^ Traditional therapies, encompassing anti-arrhythmic medications, surgical interventions, ablation, cardioversion, and medical devices, face limitations in efficacy and applicability and lead to notable adverse effects.^4,5^ While traditional CA care offers health benefits, it also incurs significant financial costs.^6^ Recognizing these limitations, there is a growing interest in exploring supplemental interventions that are both effective and cost-efficient while ensuring safety.^7^

The World Health Organization lists 31 symptoms, disorders, and diseases, including heart discomfort, arrhythmias, and hypertension, for which acupuncture has been studied extensively.^8^ Controlled research has shown acupuncture’s efficacy in treating various conditions,^9^ rooted in traditional Chinese medicine’s (TCM’s) historical use for palpitations, a common manifestation of arrhythmia.^10^ Current evidence supports acupuncture’s potential as a supplementary intervention for cardiovascular ailments, influencing cardiac rhythm through a neurological pathway that modulates sympathetic outflow.^11^ The neural mechanism forms the basis for acupuncture’s cardiovascular impacts, including blood pressure reduction, relief of myocardial ischemia, and—as demonstrated in this paper—the mitigation of ventricular arrhythmias associated with ischemia.^11^ Further research is needed to fully understand how acupuncture may reduce ischemia and arrhythmias, possibly by enhancing myocardial oxygen demand.

In the early 1990s, preliminary studies suggested that acupuncture may improve angina symptoms in individuals with ischemic heart disease, particularly those who are treatment-sensitive who show vasodilation during electroacupuncture.^12^ Experimental investigations on feline subjects with demand-induced ischemia instrumentation supported these clinical observations, revealing that acupuncture reduces myocardial ischemia by decreasing oxygen demand, particularly neutralizing increases in blood pressure during organ stimulation.^12^ The efficacy of acupuncture is closely linked to the precise choice of acupoints, a principle well acknowledged in TCM, where specific acupoints are recognized for distinct therapeutic advantages.^13–15^

Acupuncture significantly impacts therapeutic outcomes with its diverse components, such as acupoint selection, frequency, and needle retention. Yet, substantial variability in acupuncture parameters across clinical trials may lead to inconsistent results. Identifying optimal acupuncture parameters for CA management is crucial. CAs are commonly managed with anti-arrhythmic medications, such as β-blockers, calcium channel blockers, and sodium channel blockers. The choice between acupuncture and pharmacological intervention should consider patient-specific factors, including the type and severity of the arrhythmia, underlying medical conditions, and patient preferences.^1–5^ As an alternative therapy, acupuncture offers potential benefits, including minimizing adverse effects and promoting overall health.^3–5^ Controlled clinical trials are crucial to assess acupuncture’s efficacy in managing CAs and to establish its place alongside conventional pharmaceutical therapies. To address this, we performed an umbrella review of systematic reviews and meta-analyses meeting specified criteria. This study aims to compare acupuncture and pharmacological treatments, addressing safety and effectiveness concerns and offering practical recommendations for future clinical practices and trials in this field.

## Methodology

This umbrella review of systematic reviews and meta-analyses was developed following the Preferred Reporting Items for Systematic Reviews and Meta-analyses and Cochrane Collaboration Handbook guidelines.^16,17^

### Search strategy

To ensure a comprehensive search, we systematically explored relevant literature databases, including PubMed, the Cochrane Library, Embase, Scopus, and the Web of Science. The search strategy incorporated an extensive set of keywords and Medical Subject Headings (MeSH) to guarantee the inclusivity of pertinent studies. Keywords included variations of “atrial fibrillation,” “AF,” “acupuncture,” “traditional Chinese medicine,” “needle therapy,” “pharmacological treatment,” “drug therapy,” “medications,” “antiarrhythmic agents,” “rhythm control,” “cardioversion,” “anticoagulation,” and “randomized controlled trials.” Boolean operators “AND” and “OR” were strategically employed to effectively refine and broaden the search; for instance, “atrial fibrillation AND acupuncture AND pharmacological treatment” narrowed the focus to studies comparing these two interventions, while “atrial fibrillation OR AF OR rhythm control” was used to expand the search within each concept to ensure a comprehensive search. The detailed search strategy is summarized in **Supplementary Table S1**. The literature search was conducted independently by two researchers to avoid any selection bias. Consensus resolved disagreements, but a third researcher was consulted if discrepancies persisted.

### Study inclusion and exclusion criteria

#### Inclusion criteria

This umbrella review encompassed high-quality systematic reviews and meta-analyses that have examined the comparative effectiveness of acupuncture and pharmacological interventions in treating atrial fibrillation (AF) and included studies focused on adult participants aged ≥18 years diagnosed with AF. Reviews that investigated acupuncture as a treatment modality for AF were included. Additionally, studies that assessed the effectiveness of pharmacological interventions for AF, such as medications used for rhythm control, rate control, and anticoagulation, were also incorporated to facilitate a comprehensive comparative analysis. Separately, we also included meta-analyses directly comparing the efficacy and safety of acupuncture with pharmacological interventions for treating AF. Finally, we considered studies that reported relevant clinical outcomes, including but not limited to rhythm control (eg, conversion to sinus rhythm), rate control (eg, heart rate [HR] management), quality-of-life measures, safety, adverse events (AEs) associated with interventions, and cardiovascular events like stroke or myocardial infarction.

#### Exclusion criteria

Primary studies, conference abstracts, letters, editorials, and reviews not meeting the systematic review and meta-analysis criteria were excluded. Reviews on pediatric populations (participants <18 years old) or animal models were excluded. Studies examining interventions unrelated to acupuncture or pharmacological treatments for AF were not included. Reviews that did not directly compare acupuncture with pharmacological interventions in the context of AF were excluded. Studies lacking relevant clinical outcomes or reporting incomplete data were not considered for this umbrella review.

### Data extraction

Key data elements extracted included publication details (title, authors, publication year, and source), study characteristics (design, inclusion criteria, number of primary studies included), participant characteristics (age range, sex distribution), descriptions of acupuncture and pharmacological interventions (techniques, frequency, duration, medications, doses, administration), outcome measures (mortality, reduction in the recurrence of supraventricular tachycardia [SVT], reduction in ventricular premature beat [VPB] burden, reduction in the paroxysms of AF/atrial flutter, and mean HR post-treatment), effect measures used in the comparisons (eg, risk ratios [RRs], mean differences [MDs]), and details of the quality assessment methodology and results.

### Assessment of risk of bias

Two researchers independently rated the methodological quality of the included reviews and meta-analyses using the AMSTAR 2 tool. AMSTAR 2 is an instrument designed to critically assess the risk of bias in systematic reviews that consists of 16 domains referring to relevant methodological aspects, which are answered with a “yes,” “no,” “cannot answer,” or “partial yes.” The overall quality of the studies was categorized as follows: high, moderate, low, or critically low.^18^ Moreover, we assessed the risk-of-bias (quality) of the RCTs included in the individual meta-analysis using the Cochrane Collaboration risk-of-bias tool, which evaluates eight potential sources of bias, including random sequence generation, allocation concealment, blinding of participants, evaluator and outcome assessments, incomplete outcome data, missing data, and other.^19^

The certainty of evidence and strength of recommendations from meta-analyses were assessed using the Grading of Recommendations Assessment, Development, and Evaluation (GRADE) method.^20^ This tool provides a system rate with four categories, as follows: “high” when the systematic review or meta-analysis includes at least two high-quality primary studies, “moderate” when it has at least one high-quality or two moderate-quality primary studies, “low” when it contains only moderate-quality and/or inconsistent results studies, and “very low” when no medium- to high-quality studies were identified on this topic. Our starting point was “high,” and this grading decreased when we detected risk of bias, inconsistency of results (ie, I^2^ > 50%), indirectness of evidence (ie, differences in intervention), imprecision (ie, 95% confidence interval [CI] includes 1.0), or publication bias (asymmetry in funnel plot). Additionally, the rating was increased if there was a significant intervention effect, in case of a dose–response relationship, or if all plausible biases would decrease the magnitude of the intervention effect.^20^ Two researchers independently conducted the GRADE assessment with discussion and agreement for discrepancies.

### Statistical analysis

All statistical analyses and power calculations were performed using the Comprehensive Meta-analysis Software (CMA) version 4 (Biostat, Englewood, NJ, USA) and Review Manager version 5.4.1 (Cochrane, England, UK). We recalculated the effect sizes as RR values for each categorical outcome with corresponding 95% CIs using the DerSimonian and Laird random-effects model. For continuous data, we calculated the MD. *P* < .05 was considered statistically significant in two-sided tests. The heterogeneity between study associations was assessed using the I^2^ statistic.^21^ Sensitivity analyses were conducted to evaluate the robustness of summary estimates and to detect whether any individual study accounted for a large proportion of heterogeneity. Egger’s regression asymmetry test was used to calculate the evidence of small-study effects.^22^ Therefore, *P* < .05 was considered to indicate proof of small-study effects. We also assessed “*P*-hacking”^23^ and publication bias by visualizing funnel plots, trim, and fill analysis.

### Ethical considerations

As this umbrella review relied solely on previously published systematic reviews and meta-analyses, it did not involve collecting or analyzing primary data from human participants. Therefore, this study did not require ethical review board approval or collection of patient consent.

## Results

### Study selection

A total of 30 systematic reviews and meta-analyses were initially identified, with duplicate entries being excluded. After comprehensively examining complete texts, we ultimately integrated three systematic reviews and meta-analyses,^24–26^ which collectively included data from 27 randomized controlled trials (RCTs).^27–51^ **Table 1** provides a concise overview of the critical attributes of the meta-analyses incorporated in this study.

### Risk of bias of included studies

**Supplementary Figure S1:**
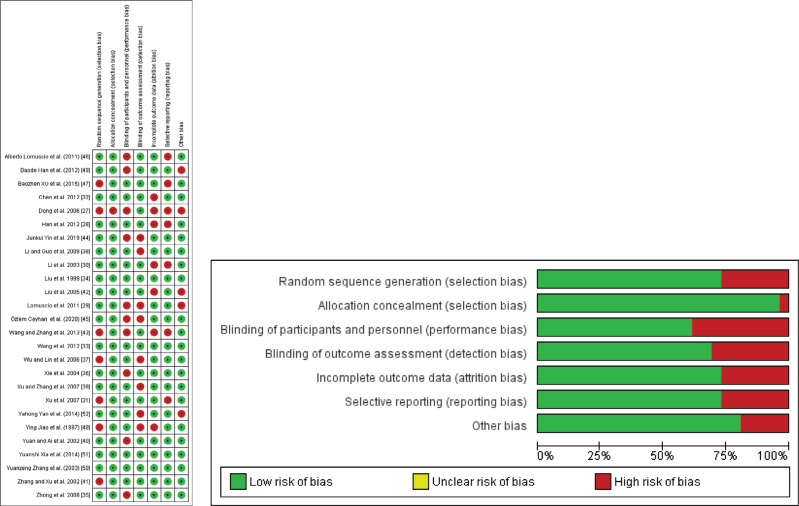
Cochrane risk-of-bias assessment for individual randomized controlled trials.

The AMSTAR 2 methodological quality ratings for the three systematic reviews and meta-analyses are presented in **Supplementary Table S2**. All three studies obtained a moderate-quality grade. According to the findings presented in **Supplementary Table S3**, the GRADE assessment revealed that the reviews included in our study exhibited a varying degree of certainty, ranging from moderate to high. The quality assessment of individual RCTs was done using the Cochrane risk-of-bias tool, which showed trials had a moderate-to-low risk of bias, as displayed in **Supplementary Figure S1**.

### Synthesis of results

#### Efficacy outcomes

The efficacy outcomes included a reduction in the recurrence rates of SVT, VPBs, and AF/atrial flutter in response to treatment as well as post-treatment HR.

#### Paroxysmal supraventricular tachycardia

**Figure 1:**
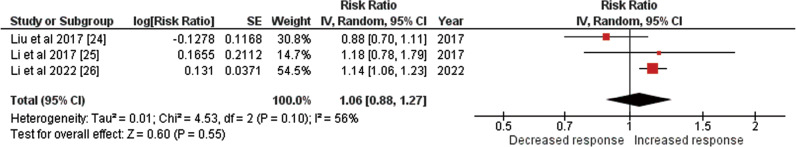
Reduction in the recurrence of paroxysmal supraventricular tachycardia. A pooled analysis of three systematic reviews and meta-analyses revealed a marginal advantage for acupuncture over standard pharmaceutical therapy in the treatment of paroxysmal supraventricular tachycardia. *Abbreviations:* CI, confidence interval; IV, inverse variance; SE, standard error.

The study incorporated three systematic reviews and meta-analyses, each providing data on paroxysmal SVT’s response to treatment. The pooled analysis demonstrated that acupuncture was associated with a marginally greater reduction in the rate of recurrence of paroxysmal SVT when compared to standard pharmaceutical therapy (relative risk, 1.06; 95% CI, 0.88–1.27; I^2^ = 56%; *P* = .55), as depicted in **Figure 1**. Nevertheless, it is crucial to acknowledge that the above findings did not achieve statistical significance, as indicated by *P* = .55. Additionally, the 95% CI for the relative risk encompassed the null value, denoted as 1.

#### Ventricular premature beats

**Figure 2:**
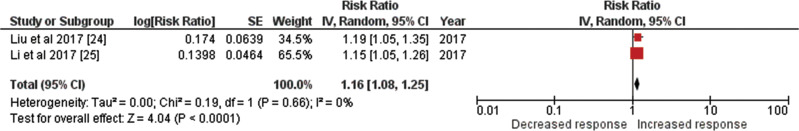
Reduction in the ventricular premature beat burden. A combined analysis of two studies demonstrated a significantly higher reduction in ventricular premature beat burden with acupuncture treatment compared to pharmacological therapy. *Abbreviations:* CI, confidence interval; IV, inverse variance; SE, standard error.

Data on VPBs’ response to treatment were available in two of the three studies. A combined analysis indicated a significantly greater reduction in the VPB burden associated with acupuncture treatment when compared to pharmacological therapy (relative risk, 1.16; 95% CI, 1.08–1.25), with no significant heterogeneity (I^2^ = 0%) and a highly significant *P* value (*P* < .0001), as illustrated in **Figure 2**.

#### Atrial fibrillation/atrial flutter

**Figure 3:**
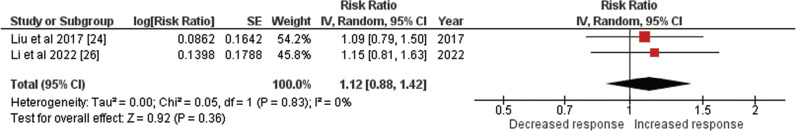
Reduction in the paroxysms of for atrial fibrillation/atrial flutter. Combined analysis of two studies revealed a higher reduction in the paroxysms of atrial fibrillation/atrial flutter with acupuncture treatment compared to pharmacological therapy. *Abbreviations:* CI, confidence interval; IV, inverse variance; SE, standard error.

Among the three studies, data regarding the response of AF/atrial flutter to treatment were available in two. The combined analysis indicated that acupuncture treatment is linked to a greater rate of reduction in the paroxysms of AF/atrial flutter when compared to pharmacological therapy (relative risk, 1.12; 95% CI, 0.88–1.42), with no substantial heterogeneity (I^2^ = 0%) and *P* = .36, as depicted in **Figure 3**. However, it is worth noting that these findings did not reach statistical significance, as suggested by the non-significant *P* value.

#### Post-treatment HR

**Figure 4:**
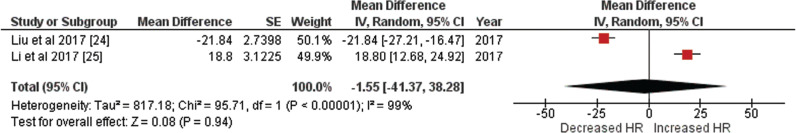
Post-treatment heart rate. A comprehensive analysis of two studies indicated a greater decrease in heart rate post-treatment with acupuncture therapy compared to pharmaceutical treatment. *Abbreviations:* CI, confidence interval; IV, inverse variance; SE, standard error.

Two studies provided data regarding HR post-treatment. The comprehensive analysis revealed that acupuncture therapy resulted in a greater decrease in HR toward normality in comparison to pharmaceutical treatment (MD, −1.55; 95% CI, −41.37 to 38.28). However, there was a notably high level of heterogeneity (I^2^ = 99%), and *P* = .94 indicated that these results did not achieve statistical significance, as shown in **Figure 4**. The high heterogeneity observed in this analysis may be attributed to several potential factors. These factors could include patient characteristics, differences in acupuncture techniques and protocols, diverse pharmacological treatments used in the control groups, and inconsistencies in outcome measurement and reporting across the studies included in the analysis. These above factors could have had a role in the extensive variability of outcomes, posing a significant challenge in formulating definitive conclusions based on the aggregated study.

#### Safety outcomes

Four trials reported AEs. The study by Zhang et al.^47^ showed that the acupuncture group exhibited no AEs. However, in the amiodarone group, one patient experienced hypotension, and two patients experienced episodes of vomiting. These AEs were effectively addressed with symptomatic therapy, allowing the patients to continue participating in the trial. Significantly, a greater incidence of AEs was reported in the cohort receiving pharmaceutical treatment compared to the cohort receiving acupuncture.

Two previous trials^32,45^ documented adverse outcomes linked to amiodarone administration by oral or intravenous routes. These AEs encompassed many symptoms, including loss of appetite (anorexia), rapid HR (sinus tachycardia), impairment in the electrical conduction system of the heart (atrioventricular block), low blood pressure (systolic blood pressure of <90 mmHg), and the act of expelling stomach contents forcefully (vomiting). In one particular investigation,^44^ it was seen that acupuncture as a standalone intervention did not result in any unfavorable occurrences like bleeding, hematoma, infection, pain, or vasovagal reactions. However, a separate study^45^ documented events of red swelling and pruritic skin associated with using catgut at acupoints.

### P-hacking, publication bias, and small-study effect

**Supplementary Figure S2:**
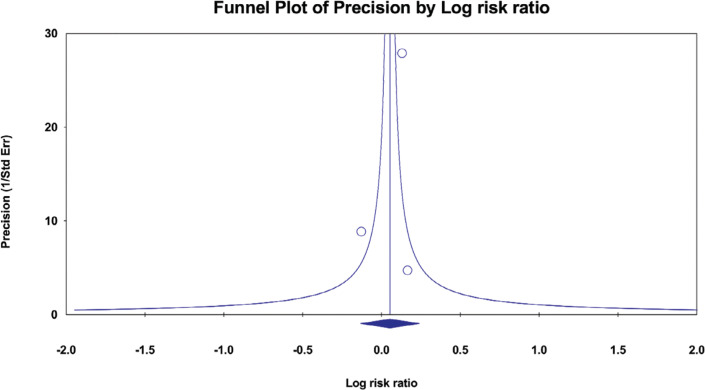
Funnel plot of outcome paroxysmal supraventricular tachycardia. The funnel plot shows no risk of publication bias. *Abbreviation:* Std Err, standard error.

The absence of evidence of *P*-hacking in our research implies that the results were not subject to manipulation to get a predetermined outcome. Notably, our assessment of publication bias was limited to more than one of the outcomes. This constraint occurs because a minimum of three studies are necessary to conduct a comprehensive analysis using a funnel plot. We could not thoroughly assess publication bias for the remaining outcomes, as each of them was only reported by two studies. In examining the reduction in the recurrence of paroxysmal SVT, we conducted a thorough analysis using sufficient studies to facilitate a funnel plot analysis. Our findings revealed that the funnel plot exhibited a symmetrical distribution of data points. The observed symmetry in the data implies the absence of publication bias, as demonstrated in **Supplementary Figure S2**. We also assessed small-study effects using Egger’s regression asymmetry test. The findings of our investigation revealed *P* values (.639) that were >.05, suggesting a lack of substantial evidence for small-study effects.

## Discussion

CAs are marked by irregular heartbeats, and types include premature beats, SVT, ventricular arrhythmia, conduction block, and bradyarrhythmia.^24^ Acupuncture, rooted in TCM, is gaining attention for its potential physiological effects on arrhythmias. Recent studies suggest its impact on the autonomic nervous system, modulating sympathetic and parasympathetic functions.^25^ Focused on specific acupoints, acupuncture activates brain circuits, particularly in the hypothalamus, midbrain, and medullary networks, regulating sympathetic outflow. This neurological regulation influences blood pressure reduction, relief from myocardial ischemia, and decreased ventricular arrhythmias linked to ischemic events.^25^ In Western medicine, Neiguan acupoint acupuncture has gained recognition for managing symptoms during transcatheter arterial chemotherapy. Experimental evidence indicates its effectiveness in alleviating palpitations and chest tension, reducing plasma endothelin levels, and modifying electrocardiographic signs related to myocardial ischemia.^25^ Bilateral acupuncture at Neiguan points is suggested to modulate the autonomic nervous system by influencing the firing rate of the amygdala, highlighting its potential anti-arrhythmic effects.^24,25^ Our study covered efficacy and safety measures for paroxysmal SVT, VPBs, AF/atrial flutter, post-treatment HR, and AEs.

The recurrence rate of paroxysmal SVT has not substantially improved with acupuncture compared to standard medical treatments.^24,25^ However, a study on acupuncture’s influence on HR variability, a metric tied to cardiovascular well-being, showed positive outcomes.^51^ While acupuncture may offer relaxation and tension relief, it should not replace primary interventions for severe cardiovascular issues, such as SVT.^25,26,51^ Regarding VPBs, Ning et al. found that combining acupuncture with TCM significantly reduced premature beats within 24 h.^52^ The study reported a standard MD of −10.55 (95% CI, −14.61 to −6.49), with a highly significant *P* value (<.00001). This suggests a potential therapeutic advantage of integrating acupuncture and TCM in treating the examined condition.

The use of early acupuncture for AF initially showed superior efficacy in restoring sinus rhythm when compared to using anti-arrhythmic medications. However, evolving therapeutic protocols led to a reduction in acupuncture’s anti-arrhythmic advantages, coupled with an increase in extracardiac adverse effects from the anti-arrhythmics. The combined use of anti-arrhythmics and acupuncture emerged to mitigate side effects and enhance overall effectiveness. In a study by Liu et al.,^24^ traditional acupuncture alone did not significantly surpass oral or intravenous amiodarone. Conversely, combining intravenous deslanoside with conventional acupuncture, as per Han et al.,^28^ showed a more pronounced augmenting effect on the response rate. The study by Lomuscio et al.^29^ supported conventional acupuncture as a standalone therapy, reducing AF recurrence post-electrical conversion for up to 3 months. However, this impact diminished at 6- and 9-month intervals. The investigation by Xu et al.,^31^ comparing acupuncture and amiodarone in paroxysmal AF, recorded significantly higher rates of sinus rhythm restoration and shorter cardioversion times in the acupuncture group.

Our investigation revealed that, compared to medication, acupuncture therapy significantly reduces HR, bringing it closer to the normal range. Li et al.^30^ documented a substantial decrease in HR (MD, −21.84; 95% CI, −27.21 to −16.47) during a 30-min acupuncture session within the conventional acupuncture group. Additionally, the study by Dong et al.^27^ showed no notable improvement in the response rate of paroxysmal SVT with a single 20-min acupuncture session in the conventional acupuncture-alone group compared to a group receiving intravenous propafenone (RR, 0.88; 95% CI, 0.70–1.09).

Our research holds a distinct advantage over individual studies as it systematically compiled and amalgamated data from multiple systematic reviews and meta-analyses about a specific topic. This approach provides a comprehensive and in-depth overview of the existing body of research on the subject. Nonetheless, our study is subject to certain limitations. First, this review encompassed only three studies, each yielding a limited number of baseline data (eg, acupuncture approach, treated sites, cycles of treatments, and types of needles) and outcomes. Consequently, further investigation is necessary to establish conclusions supported by a broader range of evidence, as quantification or measurement of neurohormonal markers, inflammation markers, and catecholamines after acupuncture were not examined to determine its effects on the heart and the mechanisms by which it might work, owing to the limited number of studies. Second, substantial heterogeneity in specific outcomes can be attributed to the diverse methodologies, inclusion criteria, and outcome measures adopted across the various systematic reviews. Third, there exists the possibility of double counting and inflation of the evidence base owing to the inclusion of certain primary studies in multiple systematic reviews. Finally, it is important to note that our study relies on synthesizing pre-existing data instead of generating new data.

## Conclusion

In conclusion, our study demonstrates that acupuncture is associated with a heightened reduction in the recurrence rates of VPBs, AF, and atrial flutter. However, it does not significantly impact the paroxysmal SVT recurrence rate or post-treatment HR. It is worth noting that, while acupuncture shows promise for specific arrhythmias, its effectiveness may vary across different conditions. For patients with VPBs, AF, or atrial flutter, considering acupuncture as a complementary therapeutic option could be beneficial. Nevertheless, further research and clinical assessment are essential to determine its precise role and suitability for specific CAs.

## Figures and Tables

**Table 1:** Characteristics of Included Meta-analyses and Systematic Reviews

Study and Year	Liu et al. (2017)^24^	Li et al. (2017)^25^	Li et al. (2022)^26^
**Study type**	Systematic review and meta-analysis	Systematic review and meta-analysis	Systematic review and meta-analysis
**Total no. of patients**	638	797	1234
**Patients in acupuncture group, n (%)**	320 (50.1)	404 (50.6)	—
**Patients in control group, n (%)**	318 (49.9)	393 (49.3)	—
**Type of control arm**	AADs, specifically mexiletine, propafenone, and amiodarone; sham acupuncture; or no treatment	AADs	Bed-rest for 30 min, AADs (specifically propafenone, diltiazem, amiodarone, mexiletine), Wenxin granule, sham acupuncture
**Inclusion criteria**	The inclusion criteria for this study encompassed patients diagnosed with any form of CA, which was verified through ECG or ambulatory rhythm monitoring techniques like telemetry, Holter monitoring, or event recording. No restrictions were placed on sex, age, course of the disease, or presence of other medical conditions. However, the baseline characteristics of each participant in the study were required to remain consistent. Trials encompassed investigations comparing the efficacy of CAs in isolation or combined with AADs against AAD therapy alone, sham acupuncture, or a no-treatment control group. These exclusions enabled the assessment of the supplementary or net effectiveness of CA interventions.	Patients who were diagnosed with AF, according to the 2020 European Society of Cardiology Guidelines for the Management of AF, and who had received acupuncture as a treatment. Also, the study design followed the methodology of a randomized controlled trial.	The inclusion criteria for this study allowed studies regardless of whether they adopted a blind approach or not and did not impose any language constraints. The inclusion criteria encompassed studies that enrolled patients diagnosed with diverse forms of CA and assigned them randomly to either undergo acupuncture treatment or to conventional therapeutic therapies.
**Exclusion criteria**	The study’s exclusion criteria encompassed malignant arrhythmias necessitating immediate defibrillation or associated with hemodynamic instability, specifically ventricular tachycardia and fibrillation. Furthermore, the application of acupuncture in the study must adhere to traditional meridian theory principles, thus excluding specific acupuncture modalities, including auricular acupuncture, scalp acupuncture based on cerebral cortex function, transcutaneous electrical nerve stimulation, laser acupuncture, and wrist–ankle acupuncture.	Studies conducted on patients who did not have AF, studies that did not include a control group, and papers that were duplicates or had been published repeatedly	Trials that complied with any of the following criteria were excluded from analysis: (1) studies with unreliable outcome data or post hoc analysis, (2) non-randomized controlled trials, and (3) studies on ear acupuncture or auricular needling
**Primary outcomes**	Mortality, recurrence rate, or readmission rate	Paroxysmal supraventricular tachycardia, ventricular premature beat, sinus tachycardia	Persistent and paroxysmal AF response to acupuncture
**Secondary outcomes**	Response rate, heart rate, ventricular rate, time of conversion to normal sinus rhythm, and quality-of-life score	N/A	N/A

*Abbreviations:* AAD, anti-arrhythmic drug; AF, atrial fibrillation; CA, cardiac arrhythmia; ECG, electrocardiogram; N/A, not available.

**Supplementary Table S1:** Search Strategy

Database	Search Strategy	Number of Articles Found
PubMed	(“conventional”[All Fields] OR “conventionals”[All Fields]) AND (“acupunctural”[All Fields] OR “acupuncture”[MeSH Terms] OR “acupuncture”[All Fields] OR “acupuncture therapy”[MeSH Terms] OR (“acupuncture”[All Fields] AND “therapy”[All Fields]) OR “acupuncture therapy”[All Fields] OR “acupuncture s”[All Fields] OR “acupunctured”[All Fields] OR “acupunctures”[All Fields] OR “acupuncturing”[All Fields]) AND (((“pharmacologically”[All Fields] OR “pharmacologicals”[All Fields] OR “pharmacologics”[All Fields] OR “pharmacology”[MeSH Terms] OR “pharmacology”[All Fields] OR “pharmacologic”[All Fields] OR “pharmacological”[All Fields]) AND (“therapeutics”[MeSH Terms] OR “therapeutics”[All Fields] OR “therapies”[All Fields] OR “therapy”[MeSH Subheading] OR “therapy”[All Fields] OR “therapy s”[All Fields] OR “”[All Fields])) OR ((“anti arrhythmia agents”[Pharmacological Action] OR “anti arrhythmia agents”[MeSH Terms] OR (“anti arrhythmia”[All Fields] AND “agents”[All Fields]) OR “anti arrhythmia agents”[All Fields] OR “antiarrhythmic”[All Fields] OR “antiarrhythmics”[All Fields] OR “antiarrhythmically”[All Fields]) AND (“agent”[All Fields] OR “agents”[All Fields])) OR (“amiodarone”[All Fields] OR “amiodarone”[MeSH Terms] OR “amiodarone”[All Fields] OR “amiodarone s”[All Fields]) OR (“adrenergic beta antagonists”[Pharmacological Action] OR “adrenergic beta antagonists”[MeSH Terms] OR (“adrenergic”[All Fields] AND “beta antagonists”[All Fields]) OR “adrenergic beta antagonists”[All Fields] OR (“beta”[All Fields] AND “blockers”[All Fields]) OR “beta blockers”[All Fields]) OR (“propranolol”[MeSH Terms] OR “propranolol”[All Fields] OR “propanolol”[All Fields]) OR (“metoprolol”[MeSH Terms] OR “metoprolol”[All Fields])) AND (“arrhythmias, cardiac”[MeSH Terms] OR (“arrhythmias”[All Fields] AND “cardiac”[All Fields]) OR “cardiac arrhythmias”[All Fields] OR (“cardiac”[All Fields] AND “arrhythmia”[All Fields]) OR “cardiac arrhythmia”[All Fields] OR (“atrial fibrillation”[MeSH Terms] OR (“atrial”[All Fields] AND “fibrillation”[All Fields]) OR “atrial fibrillation”[All Fields]) OR (“atrial flutter”[MeSH Terms] OR (“atrial”[All Fields] AND “flutter”[All Fields]) OR “atrial flutter”[All Fields]) OR (“tachycardia, ventricular”[MeSH Terms] OR (“tachycardia”[All Fields] AND “ventricular”[All Fields]) OR “ventricular tachycardia”[All Fields] OR (“ventricular”[All Fields] AND “tachycardia”[All Fields])) OR (“tachycardia, ventricular”[MeSH Terms] OR (“tachycardia”[All Fields] AND “ventricular”[All Fields]) OR “ventricular tachycardia”[All Fields] OR (“paroxysmal”[All Fields] AND “supraventricular”[All Fields] AND “tachycardia”[All Fields]) OR “paroxysmal supraventricular tachycardia”[All Fields]))	18
Cochrane Library	With all of the words: conventional acupuncture, cardiac arrhythmia With at least one of the words: (pharmacological therapy OR antiarrhythmic agents OR amiodarone OR beta blockers OR propanolol OR metoprolol) AND (cardiac arrhythmia or atrial fibrillation OR atrial flutter OR ventricular tachycardia OR paroxysmal supraventricular tachycardia)	7
Embase	Conventional acupuncture in All Text AND (pharmacological therapy OR antiarrhythmic agents OR amiodarone OR beta blockers OR propanolol OR metoprolol) in All Text AND (cardiac arrhythmia or atrial fibrillation OR atrial flutter OR ventricular tachycardia OR paroxysmal supraventricular tachycardia) in All Text	5

**Supplementary Table S2:** Assessing the Methodological Quality of Systematic Reviews—AMSTAR 2

References	AMSTAR 2 Items^a^																Overall Rating^b^
	1	2	3	4	5	6	7	8	9	10	11	12	13	14	15	16	
Liu et al.^24^	No	Yes	Yes	PY	Yes	Yes	No	Yes	Yes	No	Yes	No	No	Yes	No	Yes	Moderate
Li et al.^25^	Yes	Yes	Yes	Yes	Yes	Yes	Yes	Yes	Yes	Yes	Yes	Yes	Yes	Yes	No	Yes	Moderate
Li et al.^26^	Yes	Yes	No	Yes	Yes	Yes	Yes	Yes	Yes	No	Yes	No	Yes	Yes	Yes	No	Moderate
Total number of “yes” responses	2	3	2	2	3	3	2	3	3	1	3	1	2	3	1	2	

*Abbreviations:* PY, partial yes; RoB, risk of bias.

^a^AMSTAR 2 items are as follows:
Did the research questions and inclusion criteria for the review include the components of PICO (P: patient, I: intervention, C: control, O: outcomes)/PECO (P: patient, E: exposure, C: control, O: outcomes)? Did the report of the review contain an explicit statement that the review methods were established prior to the conduct of the review and did the report justify any significant deviations from the protocol? Did the review authors explain their selection of the study designs for inclusion in the review? Did the review authors use a comprehensive literature search strategy? Did the review authors perform study selection in duplicate? Did the review authors perform data extraction in duplicate? Did the review authors provide a list of excluded studies and justify the exclusions? Did the review authors describe the included studies in adequate detail? Did the review authors use a satisfactory technique for assessing the RoB in individual studies that were included in the review? Did the review authors report on the sources of funding for the studies included in the review? If meta-analysis was performed, did the review authors use appropriate methods for statistical combination of results? If meta-analysis was performed, did the review authors assess the potential impact of RoB in individual studies on the results of the meta-analysis or other evidence synthesis? Did the review authors account for RoB in individual studies when interpreting/discussing the results of the review? Did the review authors provide a satisfactory explanation for, and discussion of, any heterogeneity observed in the results of the review? If they performed quantitative synthesis, did the review authors carry out an adequate investigation of publication bias (small-study bias) and discuss its likely impact on the results of the review? Did the review authors report any potential sources of conflict of interest, including any funding they received for conducting the review?

^b^Rating overall confidence in the results of the review:
High: No or one non-critical weakness: the systematic review provides an accurate and comprehensive summary of the results of the available studies that address the question of interest. Moderate: More than one non-critical weakness (multiple non-critical weaknesses may diminish confidence in the review, and it may be appropriate to move the overall appraisal down from moderate to low confidence), the systematic review has more than one weakness but no critical flaws. It may provide an accurate summary of the results of the available studies that were included in the review. Low: One critical flaw with or without non-critical weaknesses, the review has a critical flaw and may not provide an accurate and comprehensive summary of the available studies that address the question of interest. Critically low: More than one critical flaw with or without non-critical weaknesses; the review has more than one critical flaw and should not be relied on to provide an accurate and comprehensive summary of the available studies. Adapted from Shea et al. 2017.^18^

**Supplementary Table S3A:** Grade Assessment of the Meta-analyses and Systematic Reviews Included in Liu et al. (2017)^24^

Certainty Assessment							No. of Patients		Effect		Certainty	Importance
No. of Studies	Study Design	Risk of Bias	Inconsistency	Indirectness	Imprecision	Other Considerations	Acupuncture	Pharmacological Treatment	Relative (95% CI)	Absolute (95% CI)		
**Response rate for paroxysmal SVT (follow-up: median, 60 days)**												
9	Randomized trial	Not serious	Serious	Not serious	Not serious	None	220/320 (68.8%)	260/318 (81.8%)	**RR 0.88** (0.70–1.11)	**98 fewer per 1000** (from 245 fewer to 90 more)	⨁⨁⨁◯ Moderate	Critical
**Heart rate (follow-up: median, 60 days)**												
9	Randomized trial	Not serious	Not serious	Not serious	Not serious	None	320	318	—	MD 21.84 lower (27.21 lower to 16.47 lower)	⨁⨁⨁⨁ High	Critical
**Response rate for VPBs (follow-up: median, 60 days)**												
9	Randomized trial	Not serious	Not serious	Not serious	Not serious	None	240/320 (75.0%)	150/318 (47.2%)	**RR 1.19** (1.05–1.34)	**90 more per 1000** (from 24 more to 160 more)	⨁⨁⨁⨁ High	Critical
**Response rate for A**F**/atrial flutter (follow-up: median, 60 days)**												
9	Randomized trial	Not serious	Not serious	Not serious	Not serious	None	140/320 (43.8%)	170/318 (53.5%)	**RR 1.09** (0.79–1.49)	**48 more per 1000** (from 112 fewer to 262 more)	⨁⨁⨁⨁ High	Critical

*Abbreviations:* AF, atrial fibrillation; CI, confidence interval; MD, mean difference; RR, risk ratio; SVT, supraventricular tachycardia; VPB, ventricular premature beat.

**Table S3B:** Grade Assessment of the Meta-analyses and Systematic Reviews Included in Li et al. (2017)^25^

Certainty Assessment							No. of Patients		Effect		Certainty	Importance
No. of Studies	Study Design	Risk of Bias	Inconsistency	Indirectness	Imprecision	Other Considerations	Acupuncture	Pharmacological Treatment	Relative (95% CI)	Absolute (95% CI)		
**Response rate for paroxysmal SVT (follow-up: median, 40 days)**												
11	Randomized trial	Not serious	Not serious	Not serious	Not serious	None	86/404 (21.3%)	59/393 (15.0%)	**RR 1.18** (0.78–1.19)	**27 more per 1000** (from 33 fewer to 29 more)	⨁⨁⨁⨁ High	Critical
**Heart rate (follow-up: median, 40 days)**												
11	Randomized trial	Not serious	Not serious	Not serious	Not serious	None	404	393	—	MD 18.8 higher (12.68 higher to 24.92 higher)	⨁⨁⨁⨁ High	Critical
**Response rate for VPBs (follow-up: median, 40 days)**												
11	Randomized trial	Not serious	Not serious	Not serious	Not serious	None	136/404 (33.7%)	110/393 (28.0%)	**RR 1.15** (1.05–1.27)	**42 more per 1000** (from 14 more to 76 more)	⨁⨁⨁⨁ High	Important

*Abbreviations:* CI, confidence interval; MD, mean difference; RR, risk ratio; SVT, supraventricular tachycardia; VPB, ventricular premature beat.

**Table S3C:** Grade Assessment of the Meta-analyses and Systematic Reviews Included in Li et al. (2022)^26^

Certainty Assessment							No. of Patients		Effect		Certainty	Importance
No. of Studies	Study Design	Risk of Bias	Inconsistency	Indirectness	Imprecision	Other Considerations	Acupuncture	Pharmacological Treatment	Relative (95% CI)	Absolute (95% CI)		
**Response rate for paroxysmal SVT (follow-up: median, 60 days)**												
11	Randomized trials	Not serious	Not serious	Not serious	Not serious	None	68/617 (11.0%)	45/617 (7.3%)	**RR 1.14** (1.06–1.22)	**10 more per 1000** (from 4 more to 16 more)	⨁⨁⨁⨁ High	Critical
**Response rate for AF (follow-up: median, 60 days)**												
11	Randomized trials	Not serious	Not serious	Not serious	Not serious	None	54/617 (8.8%)	35/617 (5.7%)	**RR 1.15** (0.81–1.52)	**9 more per 1000** (from 11 fewer to 29 more)	⨁⨁⨁⨁ High	Important

*Abbreviations:* AF, atrial fibrillation; CI, confidence interval; RR, risk ratio; SVT, supraventricular tachycardia.
